# Epidemiology and outcome analysis of 6325 burn patients: a five-year retrospective study in a major burn center in Southwest China

**DOI:** 10.1038/srep46066

**Published:** 2017-04-06

**Authors:** Haisheng Li, Zhihui Yao, Jianglin Tan, Junyi Zhou, Yi Li, Jun Wu, Gaoxing Luo

**Affiliations:** 1 Institute of Burn Research, State Key Laboratory of Trauma, Burn and Combined Injury, Southwest Hospital, Third Military Medical University, Chongqing, China; 2 People’s Liberation Army Hospital 59, Kaiyuan, Yunnan Province, China

## Abstract

Burns are a major cause of injury worldwide. We investigated the epidemiology and outcomes of burn patients in a major burn center in southwest China between 2011 and 2015 to provide guidance for burn prevention. Of the 6,325 included burn patients, 66.8% were male and 34.7% were 0 ~ 6 years old. The incidence of burns peaked in autumn. Scald was the most common cause of burns, which was predominant in patients aged 0 ~ 6 years. The mean total body surface area (TBSA) of burns was 13.4%, and patients with burns ≤10% TBSA comprised 64.1% of all cases. Patients with full-thickness burns accounted for 40.1% of all patients and 81.0% of operated patients; these burns were primarily caused by flame (34.8%), scald (21.0%), and electricity (20.4%). Fifty-six deaths occurred (mortality 0.9%), and risk factors included full-thickness burns, larger TBSA and older age. The median length of stay was 17 days, and major risk factors included more operations, better outcomes and larger TBSA. Our data showed that closer attention should be paid to children under 6 years old, males, incidents in autumn and scald burns to prevent burn injuries. Furthermore, individualized burn prevention and treatment measures based on related risk factors should be adopted.

Burns are a major cause of injury worldwide. The World Health Organization estimates that the lifetime incidence of severe burns is 1%^1^ and that more than 300,000 people die annually from fire-related burns worldwide^2^. In addition, the prevalence of burns is significantly higher in developing countries than in developed ones. Due to damage to the skin and other organs, burns can lead to open wounds, disability, death, major economic consequences, severe emotional and psychological complications, and economic burden. Therefore, burn patients require not only acute primary treatment but also subsequent rehabilitation, reconstruction and long-term anti-scar therapy. Although more than 90% of all burns are preventable, burns remain common and are a major public health problem^3^. To further improve the effects of preventive measures, studies are needed to investigate the epidemiology, etiology and outcomes of burn patient populations.

Most studies on burn epidemiology in China have been limited to specific types of burns, such as pediatric^4^,^5^,^6^, geriatric^7^, chemical^8^, electrical^9^, bedside stove^10^,^11^ and severe extensive burns^12^. Several studies have focused on the overall population of burn patients in different regions, including Beijing^13^, Shanghai^14^, Hong Kong^15^ and military hospitals^16^. However, predisposing factors, such as economic status, educational level, medical support and geographical and social environment, vary widely between cities and regions in China, and yet no comprehensive study of burn patients has been performed in southwest China to date.

This current study was performed in the Institute of Burn Research, Southwest Hospital of the Third Military Medical University (TMMU). This center is one of the longest operating burn centers in China and largest burn centers in the world; it has 125 inpatient beds (including 18 ICU beds) and specializes in burn care and treatment. Approximately 1,300 burn patients from southwest China are admitted to the center annually. The aim of this study was to describe the epidemiology and outcomes of burn patients who were admitted to the burn center of the Southwest Hospital in southwest China between January 2011 and December 2015.

## Results

**Figure 1:**
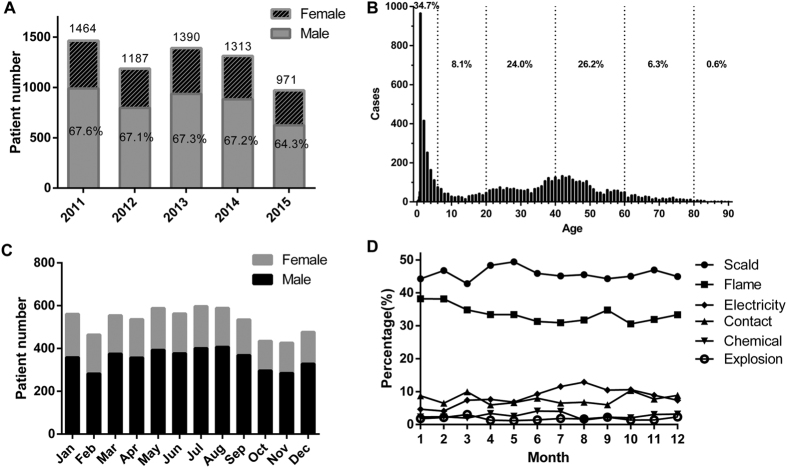
Distribution of gender, age and month. (**A**) Number of patients by gender each year. (**B**) Age distribution of all burn patients. (**C**) Number of patients by gender each month. (**D**) The distribution of burn causes by month.

From January 2011 to December 2015, 6325 burn patients were included in this study. Overall, the number of patients admitted per year exhibited a significantly decreasing trend over the five-year period, with an average admission rate of 1265 patients per year (Fig. 1A).

### Gender and age

The male-to-female ratio was 2:1 and remained constant from 2011 to 2015 (Fig. 1A). The mean age of burn patients was 27.0 years (SD: 22.6), ranging from seven days to 90 years. The three most affected age groups were of patients aged 0 ~ 6 years (preschool children, 34.7%), 41 ~ 60 years (26.2%), and 21 ~ 40 years (24.0%) (Fig. 1B).

### Time

The incidence of burns peaked in autumn (from July to September) (Fig. 1C), which is the hottest period in southwest China. The Abbreviated Burn Severity Index (ABSI)^17^, the Baux score^18^ and the Prognostic Burn Index (PBI)^19^ were calculated to quantify the extent of each burn. The ABSI, Baux score and PBI were highest in September and lowest in February (Figure S1). The proportions of different burn causes were approximately constant throughout the months, although electricity replaced contact as the third leading cause of burns from July to September, trailing only scald and flame (which remained the most common causes year-round) (Fig. 1D).

### Etiology

Table 1 illustrates the distribution of the etiology of the 6,325 patients’ burns. Scald and flame were the two most common causes of burns, accounting for 45.8% (2,893/6,325) and 33.5% (2,120/6,325) of all cases, respectively (χ^2^ = 7,415.8, P < 0.001). The male-to-female ratio differed significantly by cause (χ^2^ = 374.1, P < 0.001). The ratio was highest in electrical burns, at 8.6:1.0, followed by explosion burns (5.8:1.0), and it was the lowest for scald burns (1.3:1.0). The ratios of the other three types of burns were approximately 3:1. Furthermore, the etiology distribution differed significantly between age groups (χ^2^ = 2,360.6, P < 0.001). Scald burns were predominant in the 0 ~ 6 years age group, and flame burns were predominant in the other five age groups.

### Burn sites

As shown in Table 2, limbs were the most common burn sites, accounting for 72.1% of all admissions. The second most common site was the head, face and neck region (47.7%), followed by the trunk (43.9%). Burn sites were significantly related to etiology (χ^2^ = 1,330.5, P < 0.001); except for explosions, which primarily injured the head/face/neck, all other types of burns mainly injured the limbs. The second most common site varied by cause and was the trunk in scald burns, the head/face/neck in flame and chemical burns, and the hands in contact and electric burns.

### Burn severity

The distributions of burn severity by etiology, gender, age, burn depth and year are shown in Table 3. The ABSI, Baux score and PBI were highest for explosion burns (ABSI: 6, Baux score: 61, PBI: 50), followed by flame burns (ABSI: 5, Baux score: 52, PBI: 44), and they were lowest for scald burns (ABSI: 4, Baux score: 16, PBI: 7, P < 0.001). The ABSI, Baux score and PBI were significantly higher in males (ABSI: 5, Baux score: 41, PBI: 36) than in females (ABSI: 4, Baux score: 30, PBI: 22, P < 0.001). Patients in older age groups had higher ABSIs, Baux scores and PBIs than those in younger age groups. Furthermore, patients with full-thickness burns had a significantly higher ABSI (6), Baux score (47.5) and PBI (45) than patients without full-thickness burns (ABSI: 4, Baux score: 28, PBI: 18, P < 0.001). Patients with inhalation injury had a significantly higher ABSI, Baux score, and PBI (ABSI: 10, Baux score: 101, PBI: 66.5) than patients without inhalation injury (ABSI: 4, Baux score: 36, PBI: 30, P < 0.001). Moreover, the ABSI, Baux score and PBI were significantly higher in 2015 (ABSI: 5, Baux score: 43.5, PBI: 35.5) than in 2011 (ABSI: 4, Baux score: 35, PBI: 29.5, P < 0.01) and 2012 (ABSI: 4, Baux score: 38, PBI: 30.5, P < 0.01). The mean total body surface area (TBSA) of burns was 13.4% (SD: 16.4%, median: 8%), with a range of 0 to 100%. TBSAs of 0 ~ 10% were most frequently observed and were present in 64.1% of burn patients. In this study, five patients suffered from inhalation injury only, with a TBSA of zero.

### Full-thickness burns

In this study, patients with full-thickness burns comprised 40.1% (2,536/6,325) of all patients and 81.0% (1,924/2,374) of operated patients. As illustrated in Table 4, flame (34.8%, 882/2,536) was the most common cause of full-thickness burns, followed by scald (24.0%, 608/2,536) and electricity (20.4%, 517/2,536). The percentages of full-thickness burns differed significantly by cause and were highest in electrical burns (95.5%, 517/539) and lowest in scald burns (21.0%, 608/2,893). Full-thickness burn areas were primarily concentrated in 5% or less TBSA (67.5%, 1,709/2,536) and 1% TBSA in particular (33.6%, 851/2,536). The mortality and length of stay (LOS) of full-thickness burn patients differed significantly between different etiologies. Explosion burns had the highest mortality (14.3%) and longest LOS (45 days), and scald burns had the lowest mortality (1.8%) and shortest LOS (19 days). Compared with patients without full-thickness burns, patients with full-thickness burns were significantly older (P < 0.001), tended to be male (P < 0.001), and had a higher number of operations (P < 0.001), higher mortality (P < 0.001) and a lower improved or cured rate (P < 0.001) (Table 5).

### Length of stay

Overall, the median LOS was 17 days, ranging from 1 to 819 days. Table 6 shows the results of the multiple linear regression analysis of factors associated with LOS, and Table S1 illustrates the detailed distribution of the LOS. The natural logarithm (ln) of the LOS was calculated to meet the normality assumption. Gender, age, TBSA, full-thickness burns, inhalation injury, number of operations, outcomes, and etiology (dummy variables compared with scald burns) were included in the regression model without obvious multicollinearity (Table S2). Of these factors, having more operations prolonged LOS to the greatest extent (standardized coefficient = 0.455, P < 0.001), followed by better outcomes (standardized coefficient = 0.373, P < 0.001) and burns with a larger TBSA (standardized coefficient = 0.182, P < 0.001). Full-thickness burns, older age, and burns caused by flame, electricity, explosion, and contact were also considered risk factors for a long LOS.

### Deaths

In total, there were 56 deaths among the 6,325 patients, for a mortality of 0.9%. Logistic regression analysis was performed to screen the risk factors related to mortality. The assignment and multicollinearity analysis of the included variables are shown in Table S3, and the regression results are provided in Table 7. Our results showed that full-thickness burns had the greatest influence on mortality (OR = 16.293, P = 0.008), followed by burns with a larger TBSA (OR = 2.070, P < 0.001) and older age (OR = 1.433, P = 0.045). Moreover, a higher operation number (OR = 0.773, P = 0.002) and flame burns (OR = 0.490, P = 0.027) were protective factors for mortality. The mortality distribution is shown in Table S1, and a comparison of survivors and non-survivors is depicted in Table S4. Compared with survivors, the 56 patients who died were significantly older (41.1 ± 2.5 vs 26.8 ± 0.3 years, P < 0.001) and had significantly larger TBSA burns (72.8 ± 28.1 vs 12.8 ± 15.3, P < 0.001), more full-thickness burns (98.2% vs 39.6%, P < 0.001) and more inhalation injuries (39.5% vs 5.4%, P < 0.001). Furthermore, the non-survivors had a significantly higher ABSI, Baux score and PBI than survivors (ABSI: 14 vs 4, Baux score: 128.5 vs 38, PBI: 102.3 vs 32, P < 0.001). The distribution of mortality rate by operation number showed that five surgeries was a turning point (Table S4 and Figure S2). Before five surgeries, the mortality rate generally rose with an increase in operation number, but no deaths occurred after five surgeries.

Among the 56 deaths, 43 involved patients with >50% TBSA burns and 27 occurred among patients who suffered from an inhalation injury. The majority of patients who died (82.1%, 46/56) had at least one type of co-morbidity at admission, and the details regarding co-morbidities are shown in Table S5. The most common type of co-morbidity was respiratory disorders including inhalation injury, respiratory failure and lung trauma (34 cases). The second most common type of co-morbidity was critical illness, such as shock and sepsis (13 cases).

To evaluate the applicability of the ABSI, Baux score and PBI as predictors of mortality, a receiver operator characteristic (ROC) analysis was performed. As shown in Figure S3, the ABSI (0.962) had the greatest area under the curve (AUC), followed by the PBI (0.957) and Baux score (0.948). However, the difference was not statistically significant.

## Discussion

Investigations of burn epidemiology are crucial for evaluating the effect of current prevention measures and for adopting effective and individualized prevention approaches in the future, but they have not been conducted in southwest China. This study focuses on the epidemiological characteristics and outcomes of burn patients admitted to a major burn center in southwest China from 2011 to 2015. With the broad analysis and relatively high number of cases (compared with other studies^15^,^20^,^21^), this study’s goal was to provide guidance for burn prevention and treatment practices in southwest China.

In this study, we observed that the number of burn inpatients showed a decreasing trend over the five-year period and that the LOS was shorter in 2015 than in previous years; however, the burn severity slightly increased, and mortality did not clearly decrease. These findings suggest that more effective burn prevention and treatment measures are still needed. Consistent with previous studies^14^,^16^,^22^, our results showed that males and preschool children (under 6 years old) were at the highest risk of burn during the study period. This result might be related to the notion that males are generally more active than females and thus have a higher probability of exposure to burn risk factors. Furthermore, children under 6 years old are often unaware of danger and are curious about their surroundings. Additionally, in China, young children are often cared for by their elderly grandparents, who may have age-related physical decline or disability. Therefore, burn preventive measures with a focus on males and preschool children should be emphasized in the future. In fact, various interventions in children have achieved some success in other countries^23^,^24^.

The analysis over time suggested that the hottest season (autumn, from July to September) was associated with the highest risk of burn injury; this finding was similar to that of a study performed in Chinese military hospitals^16^ but differed from the results of a Swiss study^25^ and a study on chemical burns in east China^26^. The higher burn incidence in autumn could be due to the increased body area that is exposed, the high risk of fires induced by high temperatures, or the increased use of air conditioners and other electrical equipment. This inference was supported by the findings that the cold season (December, January and February) had the lowest severity of burns and that the rate of occurrence of electrical burns was highest during July to September. Moreover, although the head/face/neck region, which is often exposed to air, accounts for only 9% of the body surface area, burns at this site occurred in 47.7% of all patients. Awareness of this phenomenon should be increased in the future.

In contrast to previous studies^13^,^14^,^16^, scald was the most common cause of burn (45.8%) in our study, followed by flame (33.5%) and electricity (8.5%); this difference demonstrated that the burn etiology varied greatly by region and population. Associations between etiology and age or gender were also investigated. Our results showed that scald was predominant among the 0 ~ 6 years age group and in both males and females and that flame predominated in the other five age groups. Further analyses showed that the male-to-female ratio significantly differed between causes of burn: electrical burns had the highest ratio (8.6:1.0), explosion burns the second highest (5.8:1.0) and scald burns had the lowest ratio (1.3:1.0). This trend might be attributed to the different personnel composition in the different environments associated with each cause. Therefore, it is necessary to develop etiology-based burn prevention and education programs.

Our study confirmed previous findings that burns of less than 20% TBSA represent the large majority of burns^13^,^14^,^16^,^27^. In this study, 0 ~ 10% TBSA burns comprised 64.1% of burn cases, and 11 ~ 20% TBSA burns comprised 19.3%. This result indicates that it is important to protect against, evaluate the severity of, and provide appropriate therapy for burns with less than 20% TBSA. Full-thickness burns are considered one of the main risk factors of death and other outcomes and have been included in many burn scoring systems^28^. Therefore, full-thickness burns should be another treatment emphasis. In this study, 40.1% of all patients and 81.0% of operated patients suffered from full-thickness burns. In contrast to the causes in overall burn patients, full-thickness burns were most commonly caused by flame, followed by scald and electricity. These results may be determined by the injury mechanism. For example, 95.5% of electrical burns, 78.2% of contact burns, 41.6% of flame burns, and 21.0% of scald burns were full-thickness burns. These differences between causes underline the importance of developing etiology-based prevention and treatment strategies. Although burn patients with 5% full-thickness burn surface areas or less constituted the majority of full-thickness burn cases, full-thickness burn patients had significantly higher age, ABSIs, Baux scores and PBIs than burn patients without full-thickness burns. In fact, full-thickness burn patients also required more operations and had longer LOS than patients without full-thickness burns to achieve similar treatment outcomes. As a result, evidence-based protocols, including early, active and multiple operations, represent effective strategies for treating full-thickness burns.

The overall median LOS was 17 days, which was longer than the duration reported in previous studies in the Netherlands^29^ and Israel^30^ but shorter than the lengths reported in Brazil^31^ and Beijing^13^. In fact, nearly 10% of patients stayed in hospital for more than 60 days in this study. This might be because patients, especially severe burn patients, continued to stay in hospital for plastic surgery and rehabilitative treatments after their burn wounds had been cured. Accordingly, the LOS in this study may be longer than the actual wound treatment time. Furthermore, we found that a higher number of operations, a better outcome, full-thickness burns and older age were major risk factors for a long LOS. This result suggests that more active operations, such as shortening the interval between operations and increasing the size of the area treated in one operation, might be needed to shorten patients’ LOS. Furthermore, burns caused by flame, electricity, explosion, and contact were also perceived as risk factors for a long LOS, indicating the need for etiology-based individualized burn treatment strategies.

In this study, the mortality among burn patients was 0.9%, which was lower than the rate reported in previous studies^13^,^14^,^16^. The mortality was closely related to the severity of the enrolled burn patients and the level of burn treatment. Additionally, our results showed that full-thickness burns, burns with a larger TBSA and older age were risk factors for mortality and that a higher number of operations and flame burns were protective factors for mortality. However, non-survivors seemed to undergo more operations than survivors (Table S4). This interesting phenomenon could partly be explained by the following results. First, 62.77% (3,935/6,269) of survivors did not receive operations, whereas only 25% (14/56) of non-survivors did not undergo operations. Furthermore, the number of operations was similar between survivors who underwent operations and non-survivors who underwent operations (Median/IQR: 1/1 ~ 2 vs 2/1 ~ 3, P = 0.0856). Second, the distribution of mortality rate by operation number showed that five surgeries was a turning point (Table S4 and Figure S2). The mortality rate rose with an increasing number of operations before five surgeries, whereas no deaths occurred after five surgeries. Overall, among all the possible risk factors, a higher number of operations was a protective factor for mortality. Moreover, more attention should be paid to co-morbidities upon admission, and the most common co-morbidity was respiratory disorders, including inhalation injury. In addition to antibiotics, airway control and mechanical ventilation, more evidence-based individualized protocols targeting burn patients with high-risk factors for mortality should be developed and employed.

Numerous scoring systems are currently adopted to quantify burn severity and to assess burn outcomes. The main disagreement between these scoring systems is the risk factors that are included and the assigned weights. Combining as many different scoring systems as possible is recommended to achieve rigorous conclusions^32^. In this study, we chose three classical, widely used scoring systems with different factors: the Baux score (first described in 1961 and updated in 2010, this score includes age, TBSA and inhalation injury)^18^; the ABSI (first described in 1982 and revised in 2011, this index includes gender, age, TBSA, inhalation injury and presence of full-thickness burns)^17^; and the PBI (first described in 2002, this index includes TBSA of different burn depths and age)^19^. Although the differences between the AUC of the ABSI, Baux score and PBI were not statistically significant, the AUC of the ABSI was the highest, and the ROC of the ABSI was smoother than that of the other scoring systems (Figure S3). Thus, the ABSI may be more suitable for predicting mortality in our center than the Baux score and the PBI.

Nonetheless, the findings of this study should be interpreted with caution due to the following limitations. First, the enrolled patients did not include outpatients, whose burns are generally less severe than those of inpatients. As the largest burn center in southwest China, this center receives severe burn patients who are transferred from other hospitals. These two factors indicate that the burn severity in our study may be higher than that of the entire burn population. Second, this study only included data from our center, and therefore the findings cannot be directly generalized to the entire southwest region of China.

In summary, this is the first study to describe the epidemiology and outcomes of burn patients in a major burn center in southwest China between 2011 and 2015. Our findings showed that in the future, children under 6 years old, males, incidents occurring in autumn (from July to September), and scald burns should receive more attention to prevent burn injuries. Furthermore, individualized burn prevention and treatment strategies based on risk factors such as full-thickness burns, burns with a larger TBSA, older age, higher operation number and better outcomes should be adopted.

## Materials and Methods

### Ethical approval

This five-year retrospective descriptive study was approved by the Institutional Review Board of the Southwest Hospital, the Third Military Medical University. Informed consent was not required in this observational study.

### Source of data

Patients were enrolled using the following methods. First, all patients (n = 8783) admitted from January 2011 to December 2015 were extracted. Then, patients with non-burn-related issues (n = 2383), who were mainly hospitalized for skin scars, pressure ulcers, skin cancer, and chronic ulcer, were screened out based on their diagnosis by six authors. Subsequently, patients with repeated admissions or incomplete data were excluded (n = 75). Finally, a total of 6325 patients were included in this study. In addition, the following data were collected from electronic medical records: demographic data (ID number, admission date, discharge date, age, gender); injury-related data (etiology of burn injuries, depth and area of the burn, injured anatomic locations, associated complications); number of operations; LOS and patient outcomes. Patient outcomes were categorized into death, invalid, improved and cured according to the healing of patients’ wounds and their basic conditions when discharged from our center. These different outcomes were defined and evaluated based on the following criteria. If patients had died at discharge, their outcome was defined as “Death”. If the area and secretion of burn wounds did not decrease or had worsened after treatment, the patients’ outcome was defined as “invalid”. If the area and secretion of burn wounds had decreased but still existed after treatment, we defined this outcome as “improved”. If the burn wounds had completely healed without any residual wound area and secretion, the patients’ outcome was defined as “cured”. Based on the extracted data, three types of burn scores were calculated: the Baux score^18^ = Age + Percent Burn + 17 × (Inhalation injury, 1 = yes, 0 = no); the ABSI^17^ = gender (female = 1, male = 0) + age (0–20 = 1, 21–40 = 2, 41–60 = 3, 61–80 = 4, 80–100 = 5) + inhalation injury (yes = 1, no = 0) + full-thickness burns (yes = 1, no = 0) + TBSA (1–10% = 1, 11–20% = 2, 21–30% = 3, 31–40% = 4, 41–50% = 5, 51–60% = 6, 61–70% = 7, 71–80% = 8, 81–90% = 9, 91–100% = 10); and the PBI^19^ = % total body surface area (TBSA) of the third-degree burn + ½ × %TBSA of the deep second-degree burn + age.

### Statistical analysis

Data were primarily input and processed using Microsoft Excel 2007 (USA, Microsoft), and descriptive statistics (mean, standard deviation, median, interquartile range [IQR]) were calculated. Data analysis was performed using GraphPad Prism 6 (USA, GraphPad Software Inc.) and SPSS 19.0 (USA, IBM analytics). The Chi-square test was applied to assess significant associations between two categorical variables (frequency and percentage), even when the data in rows were ranked (death and etiology frequency in different age groups). However, the Kruskal–Wallis test was performed when data in the columns were ranked (different full-thickness burn areas in different etiologies, different number of operations in patients with and without full-thickness burns). The Mann-Whitney U test or Kruskal–Wallis test was conducted to compare two or more medians of categorical variables (ABSI, operation number, LOS), and Dunn’s test was performed to compare the two groups as post hoc tests. The t test or one-way ANOVA was used to compare two or more means of quantitative variables (% TBSA, Baux score, PBI, age), and Scheffe’s test was performed to compare all two groups as post hoc tests.

Multicollinearity among the included variables was analyzed using collinearity diagnostics prior to the regression. Multiple linear regression (stepwise regression method, entry: P = 0.05; removal: P = 0.10) was used to screen the risk factors for LOS. Multiple logistic regression (forward LR method, entry: P = 0.05; removal: P = 0.10) was used to screen the factors contributing to mortality. Details regarding the variable assignments and the multicollinearity results are shown in Tables S2 and S3. ROC curves were drawn, and AUCs were calculated for the ABSI, the Baux score and the PBI using SPSS 19.0. The difference between the AUCs of the three ROCs was analyzed using the Z test. P values < 0.05 were considered statistically significant.

## Additional Information

**How to cite this article**: Li, H. *et al*. Epidemiology and outcome analysis of 6325 burn patients: a five-year retrospective study in a major burn center in Southwest China. *Sci. Rep.* **7**, 46066; doi: 10.1038/srep46066 (2017).

**Publisher's note:** Springer Nature remains neutral with regard to jurisdictional claims in published maps and institutional affiliations.

## Supplementary Material

Supplementary Figures and Tables

## Figures and Tables

**Table 1:** Etiology distribution by gender and age.

Etiology	Scald	Flame	Contact	Chemical	Electricity	Explosion	Inhalation	χ^2^	*P Value*
Patients, n(%)	2893 (45.8)	2120 (33.5)	481 (7.6)	172 (2.7)	539 (8.5)	115 (1.8)	365 (5.8)	7515.8	<0.001
Gender								374.1	<0.001
Male, n(%)	1614 (55.8)	1562 (73.7)	329 (68.4)	137 (79.7)	483 (89.6)	98 (85.2)	274 (75.1)		
Female, n(%)	1279 (44.2)	558 (26.3)	152 (31.6)	35 (20.3)	56 (10.4)	17 (14.8)	91 (24.9)		
M:F Ratio	1.3:1	2.8:1	2.2:1	3.9:1	8.6:1	5.8:1	3.0:1		
Age (years)								2360.6	<0.001
0–6, n	1861	230	44	11	46	3	25		
7–20, n	169	203	40	10	68	22	35		
21–40, n	308	741	169	73	191	36	104		
41–60, n	430	723	170	73	209	52	166		
61–80, n	111	203	52	5	25	2	32		
81–100, n	14	20	6	0	0	0	3		

**Table 2:** Burn site distribution by etiology.

Etiology	Scald	Flame	Contact	Chemical	Electricity	Explosion	Total	χ^2^	*P Value*
Head/Face/Neck	1105	1522	81	86	121	99	3014	1330.5	<0.001
Limbs	2171	1593	258	113	329	95	4559		
Trunk	1555	847	69	62	180	66	2779		
Perineum	327	120	15	16	26	14	518		
Hip	563	299	39	33	52	27	1013		
Hands	172	363	112	31	213	18	909		
Feet	179	52	57	14	70	1	373		

**Table 3:** Burn severity analysis.

	ABSI		Baux Score		Prognostic Burn Index	
	Median, IQR	*Statistic, P value*	Median, IQR	*Statistic, P value*	Median, IQR	*Statistic, P value*
Etiology		300.1, <0.001		316.5, <0.001		357.3, <0.001
Scald	4, 3 ~ 5		16, 9 ~ 43		7, 3.5 ~ 36.5	
Flame	5, 4 ~ 7		52, 35 ~ 74		44, 28.5 ~ 60	
Contact	5, 4 ~ 6		44, 27.5 ~ 58		42.5, 27~54	
Chemical	5, 4 ~ 6		46.5, 34 ~ 62.5		43.5, 30.5 ~ 52.8	
Electricity	5, 5 ~ 6		44, 28 ~ 53		42.5, 27.5 ~ 52	
Explosion	6, 4 ~ 11		61, 39 ~ 109		50, 32.5 ~ 77	
Gender		2,611,162, <0.001		7.0, <0.001		7.6, <0.001
Male	5, 4 ~ 6		41, 16 ~ 59		36, 8 ~ 51	
Female	4, 2 ~ 5		30, 11 ~ 56		22, 5 ~ 49	
Age (years)		325.6, <0.001		2017, <0.001		4266, <0.001
0 ~ 6	3, 3 ~ 4		10.9, 7 ~ 16		4.5, 2.5 ~ 7	
7 ~ 20	3, 3 ~ 4		22, 16 ~ 30		17, 11.5 ~ 21	
21 ~ 40	5, 4 ~ 5		41, 34 ~ 49		36.5, 29.5 ~ 41	
41 ~ 60	6, 5 ~ 7		58, 51 ~ 71		52.5, 47 ~ 60	
61 ~ 80	7, 6 ~ 7		77, 69 ~ 88		73, 67 ~ 79.5	
81 ~ 100	8, 7 ~ 8		90, 87 ~ 97		88.3, 84.3 ~ 91.5	
Year		3.3, <0.001		4.2, <0.001		5.3, <0.001
2011	4, 3 ~ 6		35, 13 ~ 55		29.5, 5.7 ~ 47.5	
2012	4, 3 ~ 6		38, 12.9 ~ 56		30.5, 6 ~ 49	
2013	4, 3 ~ 6		40, 14 ~ 60		33, 6.8 ~ 51.5	
2014	5, 3 ~ 6		39, 15 ~ 57		34, 7 ~ 51	
2015	5, 4 ~ 6		43.5, 16 ~ 63		35.5, 8 ~ 54.5	
Inhalation injury		205,069.5, <0.001		43.0, <0.001		27.5, <0.001
With	10, 7 ~ 12		101, 76 ~ 126.5		66.5, 48 ~ 89.5	
Without	4, 3 ~ 6		36, 13 ~ 55		30, 6 ~ 48.5	
Burn depth		1,818,713, <0.001		24.8, <0.001		34, <0.001
Full-thickness	6, 5 ~ 7		47.5, 28 ~ 68		45, 26 ~ 63	
Partial-thickness	4, 3 ~ 5		28, 11 ~ 51		18, 4.0 ~ 43.5	

IQR: interquartile range

**Table 4:** Analysis of full-thickness burns by area and outcome.

Etiology	*Scald*	Flame	Contact	Chemical	Electricity	Explosion	Total	*Statistic, P value*
Cases, n(% of III° burns)	*608 (24.0*)	883 (34.8)	377 (14.9)	88 (3.5)	517 (20.4)	63 (2.5)	2536	1179.7, <0.001
Fraction of every cause (%)	*21.0*	41.7	78.4	51.2	95.9	54.8	40.1	1580.5, <0.001
III° Burn area % ^a^								454.8, <0.001
1	*215*	131	238	42	218	7	851 (33.6)	
2	*122*	96	54	7	73	6	358 (14.1)	
3	*64*	82	22	10	46	1	225 (8.9)	
4	*38*	65	8	1	31	0	143 (5.6)	
5	*39*	55	9	1	25	3	132 (5.2)	
6 ~ 10	*66*	145	26	7	67	13	324 (12.8)	
11 ~ 20	*30*	125	7	6	39	10	217 (8.6)	
21 ~ 50	*27*	135	7	12	13	15	209 (8.2)	
51 ~ 100	*7*	49	6	2	5	8	77 (3.0)	
Mortality, n(%)	*11 (1.8*)	25 (2.8)	3 (0.8)	4 (4.6)	3 (0.6)	9 (14.3)	55 (2.2)	59.5, <0.001
Length of stay								230.7, <0.001
Median	*19*	33	22	25.5	40	45	27	
IQR	*11* ~ *31.8*	16 ~ 62	14.3 ~ 44.8	11 ~ 54	21 ~ 67.5	25 ~ 112.5	15 ~ 52	

IQR: interquartile range

**Table 5:** Comparison of patients with and without full-thickness burns.

	With Full-thickness	Without Full-thickness	*Statistic value*	*P value*
Age (Years, mean ± SD)	34.3 ± 0.4	22.0 ± 0.4	22.1	<0.001
Gender			22.6	<0.001
Male, n(%)	1782 (70.3)	2445 (64.5)		
Female, n(%)	754 (29.7)	1344 (35.5)		
Number of operations				
0	612	3339	1,869,004	<0.001
1	994	370		
2	427	59		
3	207	12		
≥4	296	9		
Median, IQR	1, 1 ~ 2	0, 0 ~ 1	4,766,375	<0.001
Improved/cured, n(%)	2451 (96.6)	3767 (99.4)	70.1	<0.001
Mortality, n(%)	55 (2.2)	1 (0.03)	79.5	<0.001

IQR: interquartile range

**Table 6:** Multiple linear regression analysis of factors associated with length of stay.

Variables	Unstandardized coefficient		Standardized coefficient	*t*	*P*
	B	Std. Error	Beta		
Larger TBSA	0.011	0.001	0.182	18.047	<0.001
More operations	0.308	0.008	0.455	41.013	<0.001
Better outcomes	0.672	0.016	0.373	41.525	<0.001
Full-thickness burns	0.104	0.021	0.053	4.904	<0.001
Older age	0.001	0.000	0.021	2.176	0.030
Etiology					
Flame	0.088	0.021	0.043	4.300	<0.001
Electricity	0.201	0.036	0.058	5.602	<0.001
Explosion	0.178	0.064	0.025	2.799	0.005
Contact	0.082	0.035	0.023	2.325	0.020

Dependent variable: Ln(LOS), Constant = 0.580, R Square = 0.537, Adjusted R Square = 0.536, F = 812.258, P <0.001.

**Table 7:** Logistic regression analysis of risk factors related to mortality.

Variables	B	SE	OR	95% CI	*Wald*	*P*
Full-thickness burns	2.791	1.053	16.293	2.070 ~ 128.233	7.029	0.008
Larger TBSA	0.727	0.059	2.070	1.843 ~ 2.325	150.436	<0.001
Older age	0.360	0.180	1.433	1.008 ~ 2.039	4.012	0.045
More operations	−0.258	0.081	0.773	0.659 ~ 0.906	10.062	0.002
Flame burns	−0.713	0.323	0.490	0.260 ~ 0.922	4.884	0.027

Constant = −10.117, Chi-square = 320.474, P < 0.001; Cox & Snell R Square = 0.049, Nagelkerke R Square = 0.513.
