# Supplementary material for: Epidemiology and outcome analysis of 6325 burn patients: a five-year retrospective study in a major burn center in Southwest China
Source: Sci Rep. 2017 Apr 6;7:46066. doi: 10.1038/srep46066 (PMC5382583; doi:10.1038/srep46066)
Supplement: Supplementary Figures and Tables [file srep46066-s1.pdf]

Epidemiology and outcome analysis of 6325 burn patients: a five-year retrospective study in a major burn center in Southwest China

Haisheng Li[1], Zhihui Yao[1,2], Jianglin Tan[1], Junyi Zhou[1], Yi Li[1], Jun Wu[1], Gaoxing Luo[1]

[1]Institute of Burn Research, State Key Laboratory of Trauma, Burn and Combined Injury, Southwest Hospital, Third Military Medical University, Chongqing, China. [2]People's Liberation Army Hospital 59, Kaiyuan, Yunnan Province, China.

Supplementary Figures and Tables

Supplementary Figures

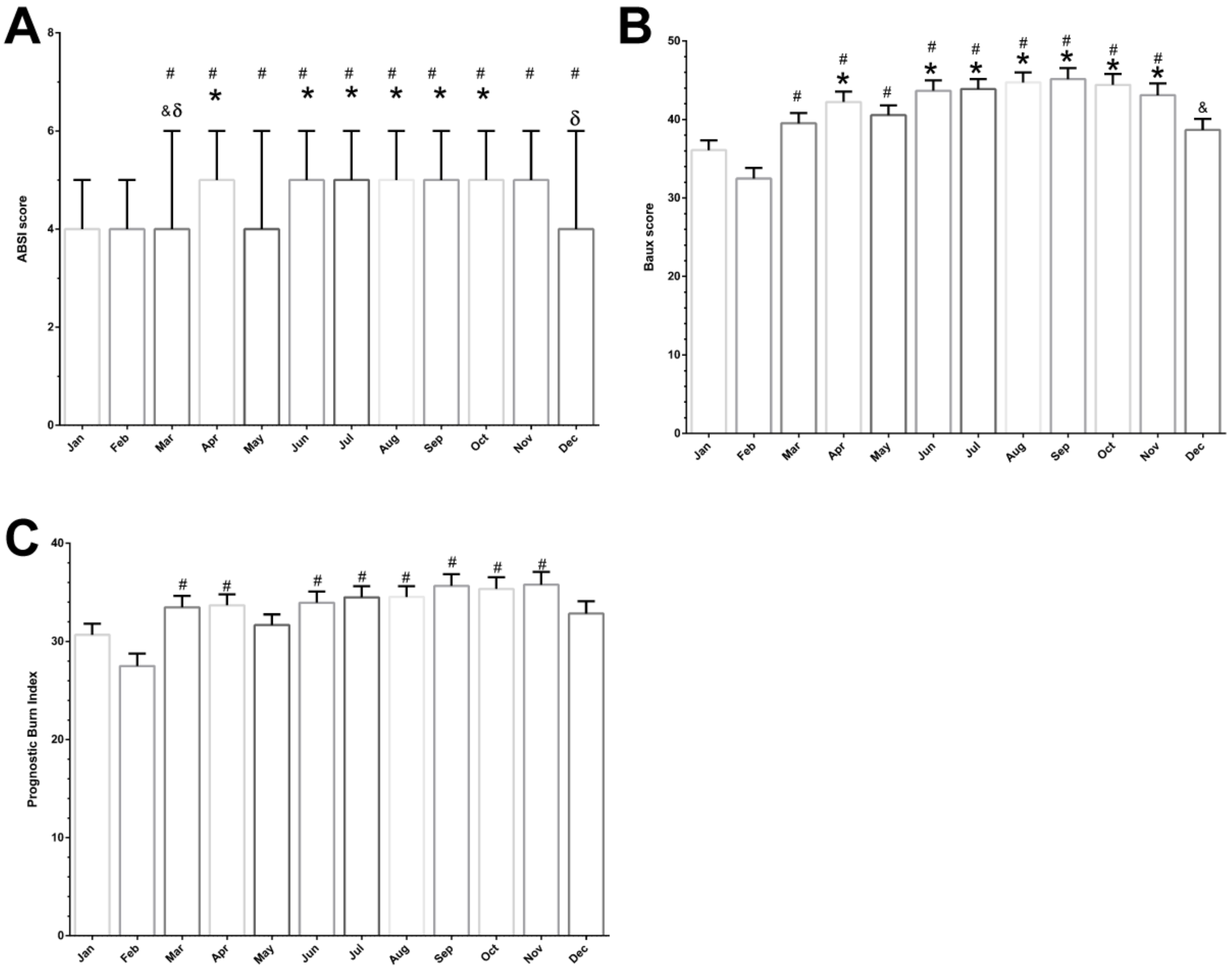

Figure S1 Distributions of burn severity by month.

A. ABSI; Kruskal-Wallis test. B. Baux scores; one-way ANOVA. C. Prognostic Burn Index; one-way ANOVA. *compared with January, $P<0.05$; $^{\#}$compared with February, $P<0.05$; $^{\&}$compared with September, $P<0.05$; $^{\delta}$compared with August, $P<0.05$; ABSI: Abbreviated Burn Severity Index.

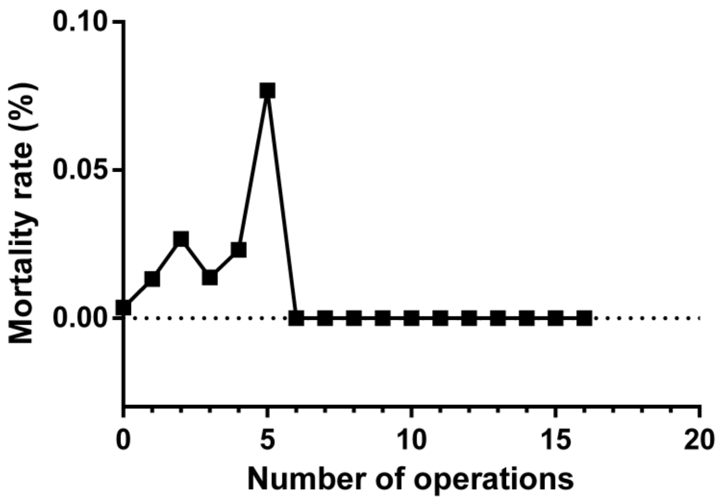

Figure S2 Distribution of mortality rate by the number of operations

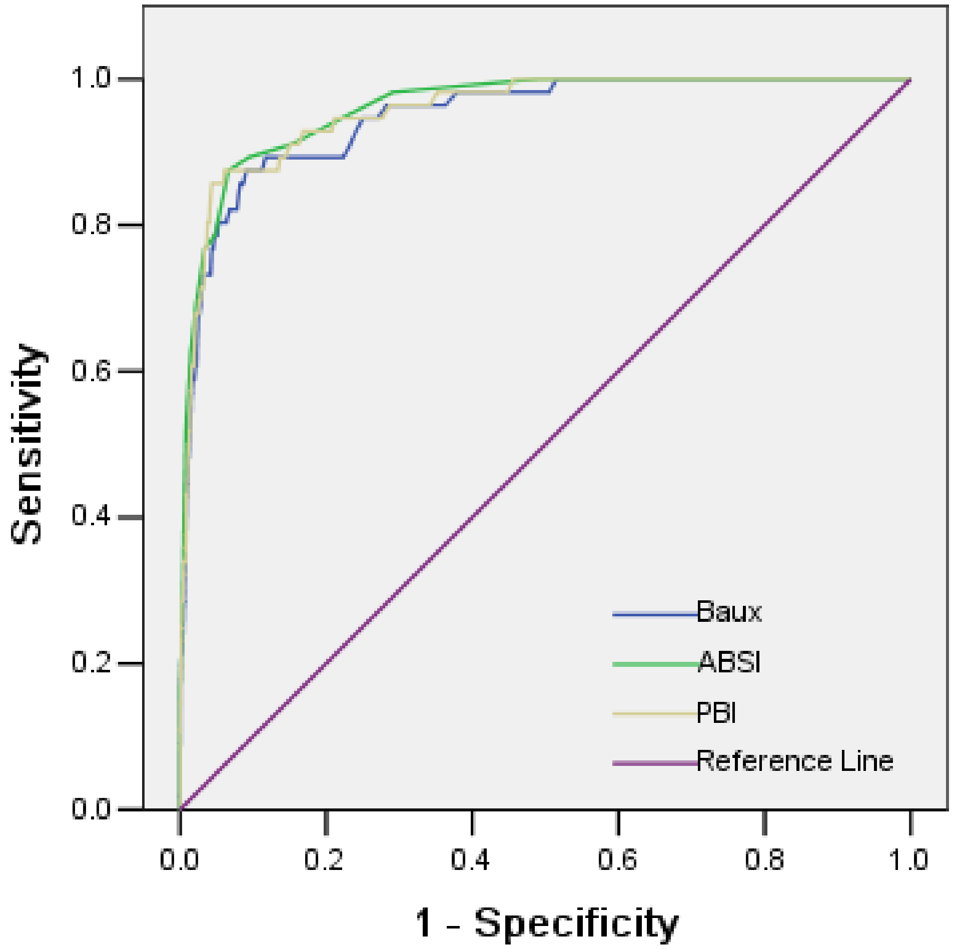

AUC of Baux score=0.948

AUC of ABSI=0.962

AUC of PBI=0.957

Figure S3 Receiver operating characteristic curves and analysis of the area under the curve to predict mortality based on 6325 patients.

Supplementary Tables

Table S1 Distribution of length of stay and mortality

	**Length of stay (days)**			**Mortality**	
	Median	IQR	*Statistic, P value*	N (%)	*Statistic, P value*
Etiology			559.8, <0.001		77.6, <0.001
Scald	13	8~23		12(0.2)	
Flame	20	11~37		25(1.2)	
Contact	20	12~39.5		3(0.6)	
Chemical	19	8~38		4(2.3)	
Electricity	40	20~66		3(0.6)	
Explosion	30	11~54		9(7.8)	
Gender			3877874, <0.001		7.5, 0.006
Male	19	10~35		47(1.1)	
Female	15	9~28		9(0.4)	
Age (years)			274.7, <0.001		29.7, <0.001
0~6	13	8~23		2(0.1)	
7~20	16	9~31		5(0.1)	
21~40	21	12~42		16(1.1)	
41~60	22	12~43		28(1.7)	
61~80	16	8~28.3		4(1.0)	
81~100	12.5	5~20.8		1(2.5)	
Year			9.6, 0.048		0.7, 0.945
2011	17	9~33		14(1.0)	
2012	18	10~34		11(0.9)	
2013	18	10~34		10(0.7)	
2014	18	10~34		13(1.0)	
2015	16	9~30		8(0.8)	
Inhalation injury			619526, <0.001		179.4, <0.001
Yes	40	18.5~93.5		27(7.4)	
No	17	9~31		29(0.5)	
Burn depth			2732290, <0.001		79.5, <0.001
Full	27	15~52		55(2.2)	
Partial	13	8~23		1(0.03)	

IQR: interquartile range

Table S2 Assignment and multicollinearity of collected factors in the linear regression analysis of length of stay

Risk factors	Variables	Evaluation	*Tolerance*	*Variance Inflation Factor*
Length of stay	Y	Ln(length of stay), consecutive variable		
Gender	X1	Male=1, Female=0	0.939	1.065
Age	X2	Consecutive variable	0.774	1.292
TBSA	X3	Consecutive variable	0.637	1.570
Full-thickness burns	X4	No=0, Yes=1	0.663	1.508
Inhalation injury	X5	No=0, Yes=1	0.735	1.360
Number of operations	X6	Consecutive variable	0.590	1.696
Outcomes	X7	Death=0, invalid=1, improved=2, cured=3	0.909	1.100
Explosion burns	X8	Dummy variables, No=0, Yes=1	0.916	1.092
Flame burns	X9	Dummy variables, No=0, Yes=1	0.659	1.517
Chemical burns	X10	Dummy variables, No=0, Yes=1	0.927	1.079
Electrical burns	X11	Dummy variables, No=0, Yes=1	0.634	1.577
Contact burns	X12	Dummy variables, No=0, Yes=1	0.755	1.324

Table S3 Assignment and multicollinearity of collected factors in the logistic regression analysis of mortality

Risk factors	Variables	Evaluation	*Tolerance*	*Variance Inflation Factor*
Death	Y	No=0, Yes=1		
Gender	X1	Male=1, Female=0	0.940	1.064
Age	X2	0~20 years=1, 21~40 years=2, 41~60 years=3, 61~80 years=4, 81~100 years=5	0.819	1.221
TBSA	X3	0~10%=1, 11~20%=2, 21~30%=3, 31~40%=4, 41~50%=5, 51~60%=6, 61~70%=7, 71~80%=8, 81~90%=9, 91~100%=10	0.664	1.505
Full-thickness burns	X4	No=0, Yes=1	0.619	1.616
Inhalation injury	X5	No=0, Yes=1	0.739	1.354
Number of operations	X6	Consecutive variable	0.626	1.598
Flame burns	X7	Dummy variables, No=0, Yes=1	0.689	1.451
Chemical burns	X8	Dummy variables, No=0, Yes=1	0.934	1.071
Electrical burns	X9	Dummy variables, No=0, Yes=1	0.647	1.547
Contact burns	X10	Dummy variables, No=0, Yes=1	0.774	1.292
Explosion burns	X11	Dummy variables, No=0, Yes=1	0.922	1.085

Table S4 Comparison between survivors and non-survivors

	Survivors	Non-survivors	*Statistic value*	*P*
Age (Years±SD)	26.8±0.3	41.1±2.5	4.7	<0.001
%TBSA			29.0	<0.001
Mean±SD	12.8±15.3	72.8±28.1		
Median, IQR	8,4~15	85,59~93.5		
Inhalation injury, n(%)	338(5.4)	27(39.5)	179.4	<0.001
Full-thickness burns, n(%)	2481(39.6)	55(98.2)	77.0	<0.001
Number of operations, n(%)			72.2	<0.001
0	3935 (99.6)	14 (0.4)		
1	1348(98.7)	18(1.3)		
2	473(97.3)	13(2.7)		
3	216(98.6)	3(1.4)		
4	127(97.7)	3(2.3)		
5	60(92.3)	5(7.7)		
6~16	110 (100)	0(0)		
Median, IQR	0,0~1	1,0.25~2	102,460	<0.001
ABSI			13,436	<0.001
Median, IQR	4,3~6	14,11~15		
Baux Score			20.4	<0.001
Median, IQR	38,14~57.5	128.5,100~144.8		
Prognostic Burn Index			19.7	<0.001
Median, IQR	32,6.5~50	102.3,85~121.1		

IQR: interquartile range

Table S5 Co-morbidity distribution among the 56 deaths

Co-morbidity type	Number involved
Shock	9
Sepsis	4
Infection	6
Inhalation injury	28
Respiratory failure	2
Lung trauma	4
Chronic obstructive pulmonary disease	3
Renal failure	2
Hypertension/ coronary heart disease	4
